# *Gangaichthys indonepalicus*, a new genus and species of miniature stygobitic earthworm eel (Synbranchiformes, Chaudhuriidae) from alluvial aquifers in the Indo-Gangetic Plain, Bihar, northern India

**DOI:** 10.3897/zookeys.1289.188089

**Published:** 2026-08-11

**Authors:** Akshay Khandekar, Tejas Thackeray, You He, Anil Mohapatra, Ishan Agarwal

**Affiliations:** 1 Thackeray Wildlife Foundation, Mumbai, 400051, India Thackeray Wildlife Foundation Mumbai India; 2 Department of Zoology, Shivaji University, Kolhapur, 416004, India Department of Zoology, Shivaji University Kolhapur India; 3 Shanghai Synchrotron Radiation Facility, Shanghai Advanced Research Institute, Chinese Academy of Sciences, Shanghai, 201204, China Shanghai Synchrotron Radiation Facility, Shanghai Advanced Research Institute, Chinese Academy of Sciences Shanghai China; 4 Estuarine Biology Regional Centre, Zoological Survey of India, Gopalpur-on-Sea, Ganjam, Odisha, 761002, India Estuarine Biology Regional Centre, Zoological Survey of India Gopalpur-on-Sea India

**Keywords:** Asia, citizen science, cytochrome oxidase, earthworm eel, hypogean, integrative taxonomy, micro-CT

## Abstract

*Gangaichthys indonepalicus***gen. et sp. nov**., is described from the Indo-Gangetic Plain, Bihar, India. This is the first stygobitic chaudhuriid and first subterranean fish from the alluvial aquifers of the Gangetic Basin and Indo-Gangetic Plain, with all three specimens collected from borewells. As the first subterranean member of the family Chaudhuriidae it is readily diagnosed from other genera and species of the family by a unique set of morphological characters linked to its stygobitic habit such as reduced eyes, the complete absence of body pigmentation, and apparent absence of ribs. In addition to its stygobitic characters, *Gangaichthys* is distinguished from all known chaudhuriid genera by the following osteological characters: neural spines laterally narrow and tapering to a point posteriorly or dorso-posteriorly, no spinelike projection on neural arch in front of neural spine; preorbital extension of ectopterygoid does not reach beyond coronoid process of dentary; metapterygoid elongate. *Gangaichthys indonepalicus***gen. et sp. nov**. may further be distinguished from all known chaudhuriid genera and species by the presence of 64 vertebrae (28 abdominal, 36 caudal), and in having a single pectoral fin-ray, six caudal fin-rays, 37 dorsal, and 35 anal fin-rays. Molecular data place *Gangaichthys indonepalicus***gen. et sp. nov**. within Chaudhuriidae, and it is > 20% divergent from two genera of the family for which there is comparative mitochondrial CO1 sequence data.

## Introduction

The family Chaudhuriidae or earthworm eels comprise seven genera and 11 species of diminutive (< 90 mm total length), elongate, freshwater fishes (Annandale 1918; Yazdani 1972; Talwar et al. 1977; Kottelat 1991; Kottelat and Lim 1994; Kullander et al. 2000; Kottelat 2000; Britz and Kottelat 2003; Britz 2010, 2016). They are found in aquatic vegetation, debris and mud in streams, swamps, ponds, and lakes. Their ecology and small size make them infrequently collected (Annandale 1918; Kottelat and Lim 1994; Kullander et al. 2000; Britz and Kottelat 2003; Britz 2016). Four genera are monotypic—*Bihunichthys monopteroides* Kottelat & Lim, 1994, *Garo khajuriai* (Talwar, Yazdani & Kundu, 1977), *Nagaichthys filipes* Kottelat & Lim, 1991 in Kottelat, 1991 and *Pillaiabrachia siniae* Britz, 2016. *Garo khajuriai* and *Pillaia indica* (Yazdani, 1972) were described from northeast India; *Chaudhuria caudata* Annandale, 1918, *Chaudhuria ritvae* Britz, 2010, *Pillaia kachinica* Kullander, Britz & Fang, 2000, and *Pillaiabrachia siniae* from Myanmar; *Chendol lubricus* Kottelat & Lim, 1994 and *Nagaichthys filipes* from Indonesia; *Bihunichthys monopteroides* and *Chendol keelini* Kottelat & Lim, 1994 from peninsular Malaysia; *Chaudhuria fusipinnis* Kottelat & Britz, 2000 in Kottelat, 2000 from Laos (Annandale 1918; Yazdani 1972; Talwar et al. 1977; Kottelat 1991; Kottelat and Lim 1994; Kullander et al. 2000; Kottelat 2000; Britz and Kottelat 2003; Britz 2010, 2016; Froese and Pauly 2025).

Annandale (1918) described the family Chaudhuriidae with the type species *Chaudhuria caudata*, and the second member of the family *Pillaia indica* was described by Yazdani (1972). Yazdani (1976) later proposed the family Pillaidae for *Pillaia indica*, now considered a part of the Chaudhuriidae (Travers 1984a, 1984b; Kottelat 1991; Britz and Kottelat 2003). A close relationship between the Chaudhuriidae and Mastacembelidae was inferred based on morphological similarity and numerous synapomorphies (Regan 1919; Annandale and Hora 1923; Yazdani 1976; Britz and Kottelat 2003); molecular data supports their sister taxon relationship, the two with Indostomidae and Synbranchidae comprising the Synbranchiformes (Harrington et al. 2024).

We came across a video posted on the social media platform TikTok in 2024 from the India-Nepal border area that showed a tiny, apparently stygobitic fish, with little melanin and reduced eyes, that had emerged from a borewell (a machine-drilled shaft used to access groundwater from underground aquifers). This prompted us to try to collect specimens of this tiny fish in 2024 and 2025 from the Indo-Gangetic Plain in Bihar, India. Many borewells later (see Materials and methods), we were able to collect one entire live specimen and two damaged specimens. Sequencing of one mitochondrial and one nuclear marker allowed us to determine that this fish belongs to the Chaudhuriidae; while high resolution micro-CT scans revealed a unique set of osteological characters. This is the first stygobitic chaudhuriid and clearly differs from all known genera and species in morphology, and we describe this as a new genus and new species, *Gangaichthys indonepalicus* gen. et sp. nov.

## Materials and methods

### Taxon sampling

We sampled more than 15 borewells by hand-pumping for approximately one week and were able to collect three specimens of the new species from two borewells. Only one specimen was intact and live, which was photographed in life using a Canon 80D DSLR camera mounted with 100 mm macro lens and two external flashes, and later euthanized using clove oil. The two dead, damaged specimens were fixed in molecular grade ethanol and subsequently stored at –20°. The intact specimen was fixed in 4% formaldehyde for ~12 hours with fins spread using a drawing brush; then washed thoroughly in water before being transferred to 70% ethanol for long-term storage. These specimens are deposited in the museum and research collection facility at the National Centre for Biological Sciences, Bengaluru (institutional code **NRC**). GPS coordinates are not provided to protect against illegal collection (available on request from the authors).

### Molecular data and phylogenetic analyses

We extracted DNA from the intact and one damaged specimen of *Gangaichthys indonepalicus* gen. et sp. nov. using the Qiagen® DNeasy Blood and Tissue Kit. We targeted fragments of two protein coding genes—mitochondrial Cytochrome Oxidase 1 (CO1) and nuclear RAG1 using the primer pairs FishCOI_F2 (TCGACTAATCATAAAGATATCGGCAC) + FishCOI_R1 (TAGACTTCTGGGTGGCCAAAGAATCA) (Ward et al. 2005) and RAG1fish_F1 (CTGAGCTGCAGTCAGTACCATAAGATGT) + RAG1fish_R1 (CTGAGTCCTTGTGAGCTTCCATRAAYTT) (López et al. 2004), respectively, for both PCR and bidirectional Sanger sequencing (outsourced to Medauxin, Bengaluru, India). We used BLAST (https://blast.ncbi.nlm.nih.gov/Blast.cgi) to determine the most similar sequences on GenBank which showed a close relationship of our sequences with representatives of Synbranchiformes. We thus assembled a dataset including representatives of the Chaudhuriidae, Mastacembelidae, Indostomidae and Synbranchidae with *Channa* spp. (Anabantiformes: Channidae) as outgroups (Table 1). Only two species of the Chaudhuriidae have CO1 sequences available and there are no comparative nuclear sequence data for the family. Sequences were aligned using default settings in ClustalW (Thompson et al. 1994) in MEGA 5.2 (Tamura et al. 2011) and translated to amino acids to ensure no premature stop codons were present. Uncorrected p-distance was calculated in MEGA 5.2 using the pairwise deletion option.

**Table 1.** Sequences used in this study with GenBank accession numbers.

**Family**	**Species**	**CO1**	**RAG1**
Chaudhuriidae	*Chaudhuria caudata*	LC190183	–
	*Chaudhuria caudata*	LC190185	–
	*Gangaichthys indonepalicus* gen. et sp. nov.	PZ760211	PZ760212
	*Gangaichthys indonepalicus* gen. et sp. nov.	PZ760211	PZ760212
	*Pillaia indica*	KY172977	–
	*Pillaia indica*	KY172978	–
	*Pillaia indica*	KY172979	–
	*Pillaia indica*	MT394417	–
	*Pillaia indica*	OK258091	–
	*Pillaia indica*	MK572486	–
	*Pillaia indica*	MK572487	–
Indostomidae	*Indostomus paradoxus*	AP004438	KF141263
	*Indostomus paradoxus*	NC_4401	KF141262
Mastacembelidae	*Macrognathus aral*	KY290061	LT577438
	*Macrognathus pancalus*	KY290044	LT577440
	*Macrognathus zebrinus*	KT944635	–
	*Mastacembelus alboguttatus*	KT944638	–
	*Mastacembelus congicus*	MK074461	LT577387
	*Mastacembelus erythrotaenia*	JQ769007	KF017115
	*Sinobdella cf. sinensis*	KT944657	–
	*Sinobdella sinensis*	MK321934	–
Synbranchidae	*Monopterus albus*	MT884625	JN979995
	*Monopterus javanensis*	MK628406	–
	*Monopterus javanensis*	MW591100	–
	*Ophichthys cuchia*	KR705867	–
	*Ophichthys cuchia*	MW315780	–
	*Ophichthys fossorius*	MW315779	–
	*Ophichthys fossorius*	MW3157791	–
	*Ophichthys indicus*	KX946707	–
	*Ophichthys indicus*	MW315781	–
	*Ophisternon aenigmaticum*	EU751859	AY359220
	*Ophisternon bengalense*	KP729589	KP738644
	*Ophisternon candidum*	MH001810	–
	*Ophisternon* sp.	JN313622	–
	*Rakthamichthys digressus*	PP818913	–
	*Rakthamichthys indicus*	MW315776	–
	*Rakthamichthys mumba*	MZ452067	–
	*Rakthamichthys roseni*	MW315777	–
	*Rakthamichthys* sp.	PP263635	–
	*Synbranchus marmoratus*	MZ051994	ON419053
Channidae (outgroups)	*Channa argus*	KC819594	LC258107
	*Channa gachua*	KJ847097	LC258409
	*Channa striata*	KP842451	LC258430

We reconstructed phylogenetic relationships with Maximum Likelihood (ML) in RaXML HPC 8.2.12 (Stamatakis 2014) for each marker and the concatenated dataset, and Bayesian inference (BI) in MrBayes 3.2.7 (Ronquist and Huelsenbeck 2003) for the concatenated dataset. The best-fitting partitioning schemes and models of sequence evolution were estimated with each gene fragment partitioned by codon position (cp) in PartitionFinder 2.1.1 (Lanfear et al. 2016) with linked branch lengths and selection based on the Bayesian information criterion (BIC) (GTR+I+G for CO1 cp1 + RAG1 cp2; GTR+G for other partitions). ML analyses applied the GTR + G model with 10 independent ML runs and support assessed through 500 thorough bootstraps. Partitioned BI analysis used two parallel runs with four chains each (three hot and one cold) with parameters unlinked, run for 1,000,000 generations, sampling every 100 generations. Convergence of BI was determined by inspecting log files in Tracer 1.7.2 (ESS >> 200; Rambaut et al. 2018; available at https://github.com/beast-dev/tracer/releases/tag/v1.7.2) and the standard deviation of split frequencies (<< 0.01), with the first 25% of trees discarded as burn-in and a consensus tree built using the sumt function. We also ran analyses dropping the third position of the mtDNA marker CO1 which did not change interrelationships (not shown).

### Morphological data

The single intact and another relatively intact specimen of *Gangaichthys indonepalicus* gen. et sp. nov. were used for all morphological work as the third specimen is fragmentary. We recorded colour pattern from live photographs and a single observer (AK) recorded morphological data under a ZEISS Stemi 305 stereo dissecting microscope. Handling and measuring specimens of different chaudhuriid species can be challenging due to their small size and often bent or contorted condition (Britz 2010) and thus only a few accurate measurements were obtained for mainly the intact specimen, taken following Kullander et al. (2000) and Britz (2010) with a Mitutoyo digital calliper to the nearest 0.1 mm. Comparative morphological data on other chaudhuriid species were taken from literature (Annandale 1918; Yazdani 1972, 1976; Talwar et al. 1977; Yazdani and Talwar 1981; Kottelat and Lim 1994; Kottelat 2000; Kullander et al. 2000; Britz and Kottelat 2003; Britz 2010, 2016). Bone terminology follows Britz and Kottelat (2003).

### Computed tomography

NRC-AA-9519 was scanned by Sunil Prabhakar in February 2025 at the electron microscope facility at the National Centre for Biological Sciences (TIFR-NCBS), Bangalore using Bruker® Skyscan 1272 (Bruker BioSpin Corporation, Billerica, Massachusetts, USA). The scan was performed without the use of contrast agents and parameters were: 38 kV, 120 µA, no filter, 1200 projections, sample half rotation, frame averaging = 2. Digital segmentation of the individual elements was conducted in CT Analyzer version 1.23.0.2+, 3D view of the sample was generated in CTvox version 3.3.1 (Bruker BioSpinCorporation, Billerica, Massachusetts, USA) and images were edited in Adobe Photoshop CS6.

## Results

### Phylogenetic relationships

**Figure 1.**
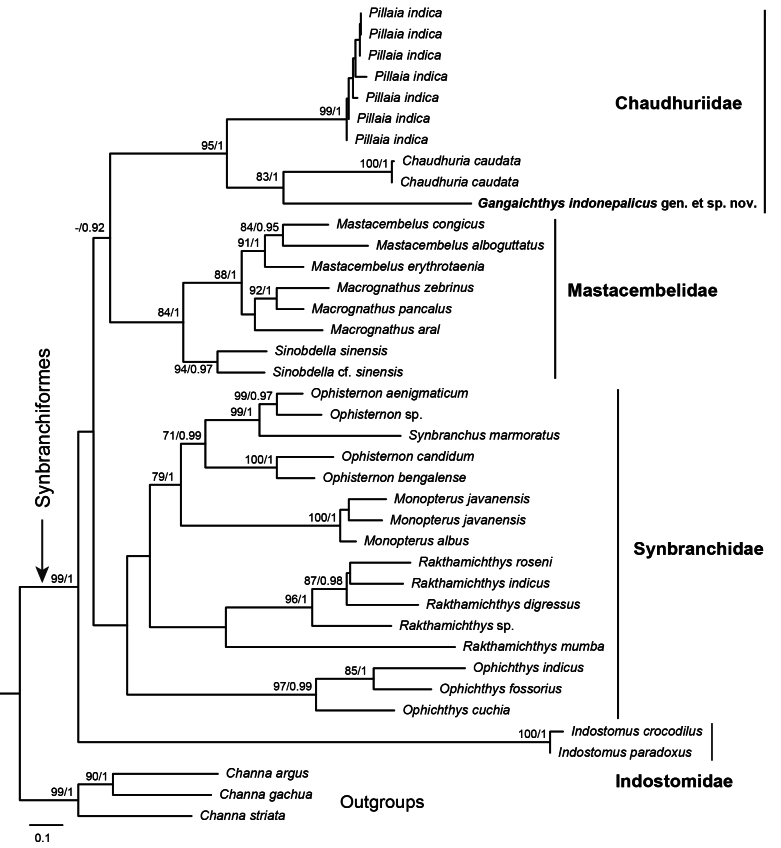
Maximum likelihood phylogeny of the Synbranchiformes. Numbers at nodes indicate bootstrap support/ posterior probability (> 70, 0.9).

Phylogenetic analyses of the concatenated dataset (Fig. 1) and individual markers (not shown) recovered the Synbranchiformes and its constituent subfamilies with high support (BS > 80, PP 1), except for the Synbranchidae which did not receive high support. A sister-taxon relationship was recovered for the Chaudhuriidae and Mastacembelidae, though with only moderate support from BI (PP 0.92) and low ML support. *Gangaichthys indonepalicus* gen. et sp. nov. is nested within the Chaudhuriidae, the family represented by only CO1 sequences for *Chaudhuria caudata* and *Pillaia indica*. The CO1 and RAG1 sequences of both specimens are identical; an average of (CO1 divergence, RAG1 in parentheses) 20.7% from *Chaudhuria caudata* and 22.2% from *Pillaia indica*; 25.4% (12.7%) from Mastacembelidae, 28.2% (13.5%) from Synbranchidae and 29.5% (17.9%) from Indostomidae.

### Systematics

#### Gangaichthys gen. nov.

Taxon classificationAnimalia

6DE857DD-BEC4-5832-BCCD-010348EEDB49

https://zoobank.org/9B4D1A6C-62A0-41FC-AD8A-D60AFDD608D8

**Figure 2.**
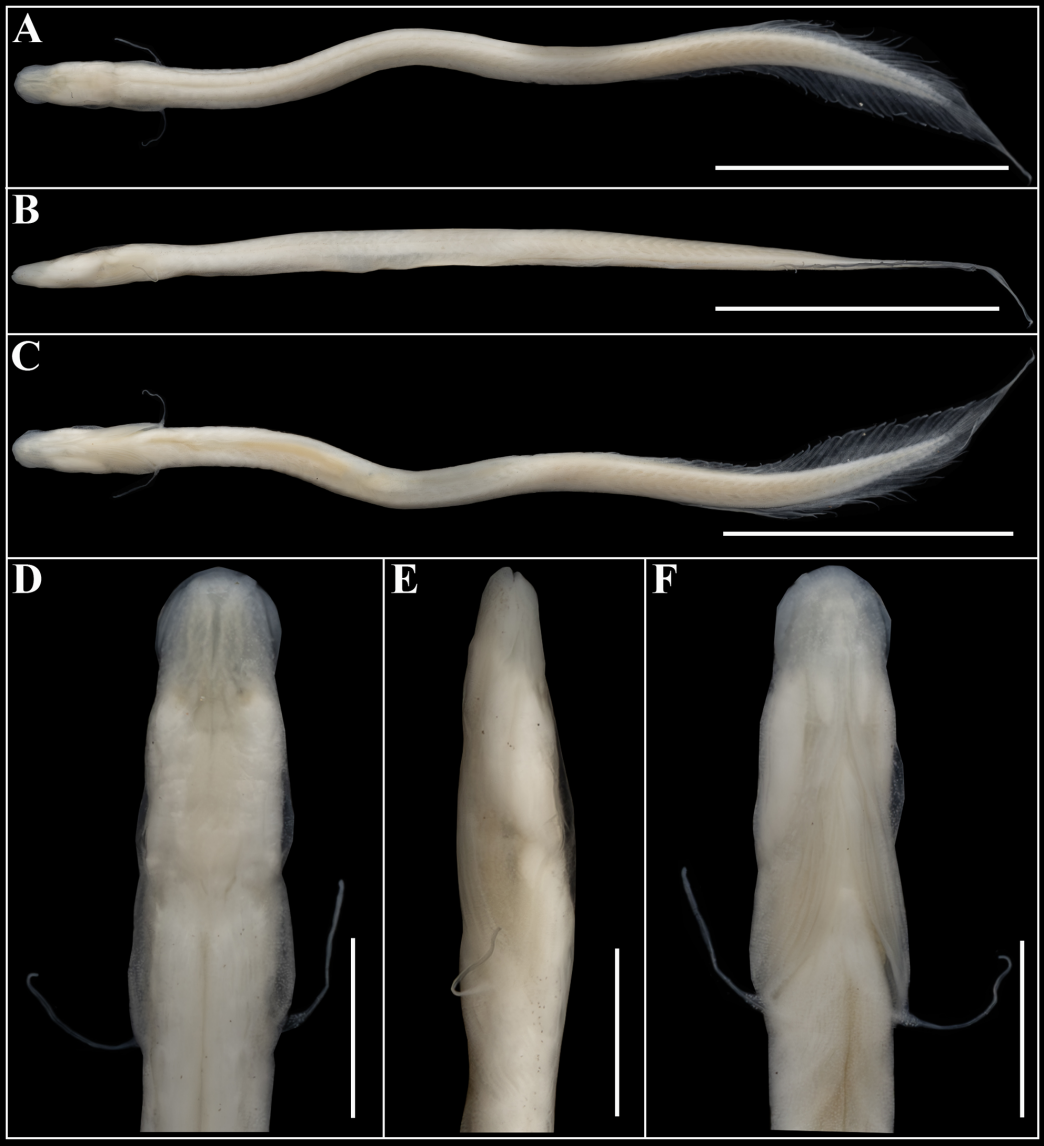
*Gangaichthys indonepalicus* gen. et sp. nov., NRC-AA 9519, holotype, 40.6 mm SL. **A**. Dorsal view of body; **B**. Lateral view of body; **C**. Ventral view of body; **D**. Dorsal view of head; **E**. Lateral view of head; **F**. Ventral view head. Photos by Akshay Khandekar. Scale bars: 10 mm (**A–C**); 2 mm (**D–F**).

**Figure 3.**
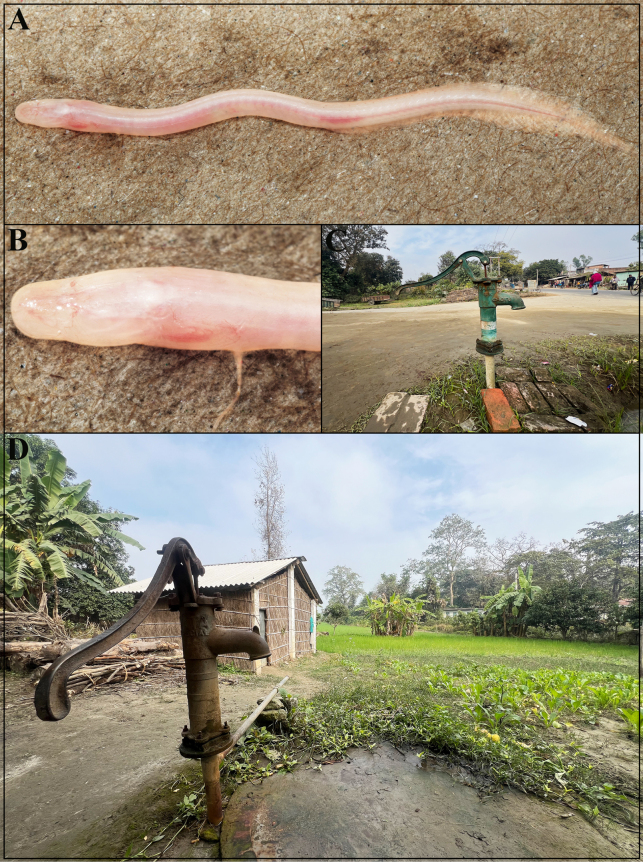
Live and habitat photos *Gangaichthys indonepalicus* gen. et sp. nov.: NRC AA 9519, holotype, 40.6 mm SL: Dorso-lateral view of: **A**. Full body; **B**. Head; **C**. Bore-well from which holotype and one (damaged) paratype was collected; **D**. Bore-well from where another damaged specimen was collected. Photos by Akshay Khandekar.

**Figure 4.**
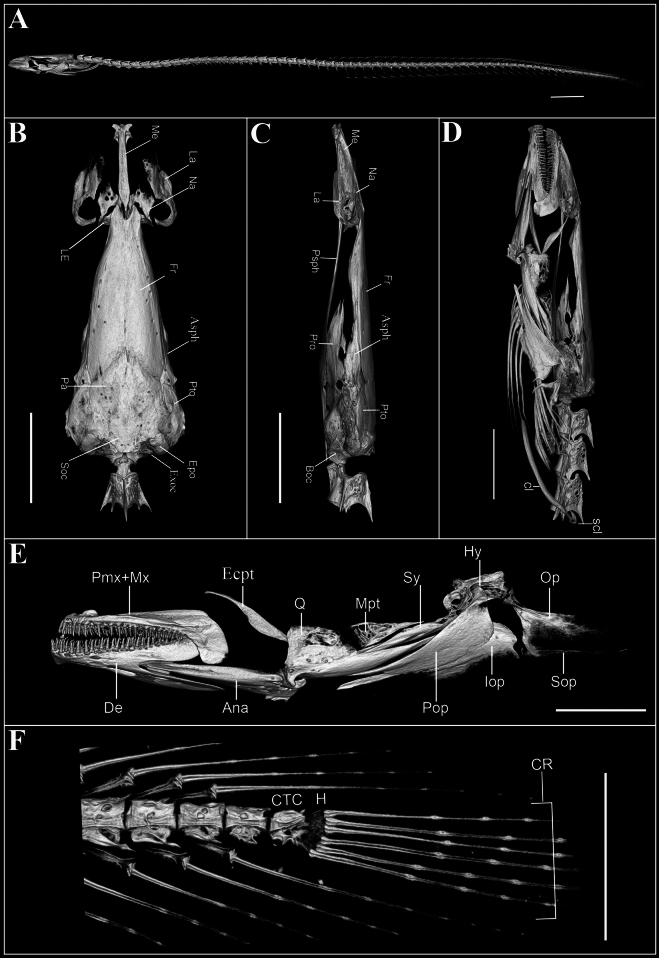
*Gangaichthys indonepalicus* gen. et sp. nov. (NRC-AA 9519, holotype) micro-CT. **A**. Whole body; **B**. Neurocranium plus first vertebra in dorsal view; **C**. Neurocranium plus first vertebra in lateral view; **D**. Head, pectoral girdle plus first three vertebrae; **E**. Jaws, suspensorium and opercular bones; **F**. Caudal skeleton. Abbreviations: Ana = anguloarticular, Asph = autosphenotic, Boc = basioccipital, Cl = cleithrum, CTC = compound terminal centrum, CR = caudal fin-rays, De = dentary, Ecpt = ectopterygoid, Epo = epiotic, Exoc = exoccipital, Fr = frontal, H = hypural, Hy = hyomandibula, Iop = interopercle, La = lacrimal, LE = lateral ethmoid, Me = mesethmoid, Mpt = metapterygoid, Na = nasal, NS = neural spine, Op = opercle, Pa = parietal, Pmx+Mx = Premaxilla plus maxilla, Pop = preopercle, Pro = prootic, Psph = parasphenoid, Pto = pterotic, Q = quadrate, Scl = supracleithrum, Soc = supraoccipital, Sop = subopercle, Sy = symplectic. Scale bars: 1 mm.

Figs 2, 3, 4; Tables 2, 3

##### Type species.

*Gangaichthys indonepalicus* sp. nov.

**Table 2.** Selected size-standardised value (original measurements in parentheses) of *Gangaichthys indonepalicus* gen. et sp. nov., / = data unavailable.

**Type**	**Holotype**	**Paratype**
Specimen no.	NRC-AA-9519	NRC-AA-9695
Standard length	40.6	36.5
In % of standard length		
Head length	13.0 (5.3)	8.9 (3.2)
Head depth	4.2 (1.7)	/
Head width	4.6 (1.8)	/
Body depth at anal fin origin	3.8 (1.5)	/
Body width	3.6 (1.5)	/
Snout length	3.8 (1.5)	1.8 (0.6)
Pre-dorsal length	49.1 (19.9)	/
Pre-anal length	46.9 (19.0)	/
Pre-anus length	45.0 (18.3)	/
Pectoral fin length	5.0 (2.1)	7.5 (2.7)
Caudal fin length	10.0 (4.1)	9.4 (3.4)

**Table 3.** Key diagnostic characters of *Gangaichthys indonepalicus* gen. et sp. nov. and other Chaudhuriids. NA = not available/unknown, A = absent.

**Species**	**Source**	**Vertebrae**			**Fin-rays**				**Anterior neural spine**	**Metapterygoid**	**extent of preorbital extension of ectopterygoid**	**premaxilla-maxilla**	**Preorbital-autosphenotic**
		**Abdominal**	**Caudal**	**Total**	**Dorsal**	**Anal**	**Caudal**	**Pectoral**					
*Gangaichthys indonepalicus* gen. et sp. nov.	Current study	28	36	64	37	35	6	1	Not expanded, tapering posteriorly	elongate	not beyond coronoid process of dentary	fused	separate
*Bihunichthys monopteroides*	Britz and Kottelat (2003)	33–35	39–41	74	39–43	41–44	1	1	Not expanded, tapering upward	? Normal	beyond coronoid process of dentary	separate	separate
*Chaudhuria caudata*	Annandale (1918); Britz (2016)	27–29	42–45	70–74	39–42	39–43	8–9	7–9	expanded, oriented upward	normal	beyond coronoid process of dentary	Separate	in contact
*Chaudhuria fusipinnis*	Kottelat (2000); Britz and Kottelat (2003)	27–29	42–47	71–74	44–48	44–48	6–7	6–10	NA	NA	NA	NA	NA
*Chaudhuria ritvae*	Britz (2010)	27–28	46–49	74–76	40–43	32–40	8	7–10	expanded, oriented upward	normal	beyond coronoid process of dentary	Separate	in contact
*Chendol keelini*	Kottelat and Lim (1994); Britz and Kottelat (2003)	39–41	25–28	66–68	43–45	41–44	4–6	14–17	expanded, oriented upward	normal	NA	Separate	in contact
*Chendol lubricus*	Britz and Kottelat (2003)	34	35	67	36–37	36–37	6	12–14	expanded, oriented upward	normal	beyond coronoid process of dentary	Separate	in contact
*Garo khajuriai*	Talwar et al. (1977); Yazdani and Talwar (1981)	NA	NA	65	40–44	37–38	12	19–20	NA	NA	NA	NA	NA
*Nagaichthys filipes*	Britz and Kottelat (2003)	25–27	28–32	54–59	29–32	29–32	4	0–4	Not expanded, tapering upward	normal	beyond coronoid process of dentary	fused	reduced, no anterior process
*Pillaia indica*	Yazdani (1972, 1976); Yazdani and Talwar (1981); Britz and Kottelat (2003); Britz (2016)	26–29	35–38	62–67	34–39	33–38	8–11	6–9	Not expanded, tapering upward	normal	beyond coronoid process of dentary	fused	reduced, no anterior process
*Pillaia kachinica*	Kullander et al. (2000); Britz (2016)	26–28	44–46	71–74	42–44	44–45	10–12	10–11	NA	NA	NA	NA	NA
*Pillaiabrachia siniae*	Britz (2016)	37	34	71	31	33	6	A	Not expanded, tapering upward	NA	NA	NA	NA

##### Diagnosis.

*Gangaichthys* gen. nov. differs from all other genera of the family Chaudhuriidae by a number of morphological characters such as complete absence of body pigmentation, reduced eyes, and apparent absence of ribs. It differs further by the combination of the following characters: 64 vertebrae in total (28 abdominal + 36 caudal); and 37 dorsal, 35 anal, a single pectoral, and six caudal fin-rays; dorsal and anal fins long, connected to caudal fin by a membrane; anterior process of the prootic evident, dorsally free from the autosphenotic; all neural spines laterally narrow and tapering to a point, oriented posteriorly or postero-dorsally; absence of anterior spinelike projection on neural arch; preorbital extension of ectopterygoid does not reach beyond coronoid process of dentary; metapterygoid elongated; a single hypural, premaxilla and maxilla fused (Tables 2, 3).

##### Comparative remarks.

The stygobitic *Gangaichthys* gen. nov. exhibits classic reductive traits associated with hypogean environments, including complete absence of body pigmentation, apparent absence of ribs, and reduced eyes. These traits are often the result of convergent evolution, driven by relaxed selection in dark, resource-poor habitats, rather than indicators of phylogenetic distinctiveness (Dahanukar et al. 2023). However, the new genus can be easily diagnosed from all other chaudhuriid genera by several previously used, osteological, morphological, and meristic diagnostic characters.

*Gangaichthys* gen. nov. and the monotypic *Bihunichthys* are similar in having a single pectoral fin; however, they can be easily distinguished from one another by the absence of anterior spinelike projection on neural arch (vs presence of spinelike projection on neural arch in front of neural spine); by having 37 dorsal, 35 anal, and six caudal fin rays (vs 39–43 dorsal, 41–44 anal and a single caudal fin ray in *Bihunichthys*); non-overlapping vertebrae counts—64 total vertebrae including 28 abdominal + 36 caudal (vs 74 total vertebrae including 33–35 abdominal + 39–41 caudal). *Gangaichthys* gen. nov. and the monotypic *Garo* are similar in the number of total vertebrae (64 vs 65 in *Garo*) but can easily be distinguished from each other in having a single pectoral, 37 dorsal, 35 anal, and six caudal fin rays (vs 19–20 pectoral, 40–44 dorsal, 37–38 anal, and 12 caudal fin rays). *Gangaichthys* gen. nov. and the monotypic *Nagaichthys* have similar number of abdominal vertebrae (28 vs 25–27 in *Nagaichthys*) and pectoral fin rays (1 vs 0–4); however, both can be easily separated from one another in having non overlapping counts of total (64) and caudal (36) vertebrae (vs 54–59 total and 28–32 caudal vertebrae); having 37 dorsal, 35 anal, and six caudal fin rays (vs 29–32 dorsal, 29–32 anal, and four caudal fin rays). The new genus and the monotypic *Pillaiabrachia* both lack ribs and have six caudal fin rays, but can be easily distinguished from each other by having a single pectoral, 37 dorsal and 35 anal fin rays (vs pectoral fin absent, 31 dorsal, and 33 anal fin rays in *Pillaiabrachia*); 64 total vertebrae including 28 abdominal and 36 caudal (vs 71 total vertebrae including 37 abdominal and 34 caudal). *Gangaichthys* gen. nov. and *Chaudhuria* share a similar number of abdominal vertebrae (28 vs 27–29 in *Chaudhuria*) and caudal fin rays (6 vs 6–9), but can be easily separated by having non overlapping counts of total (64) and caudal (36) vertebrae (vs 70–76 total and 42–49 caudal vertebrae); having a single pectoral, 37 dorsal, and 35 anal fin rays (vs 6–10 pectoral, 39–48 dorsal, and 32–48 anal fin rays). *Gangaichthys* gen. nov. and *Chendol* have similar number of dorsal (37 vs 36–45) and caudal fin rays (6 vs 4–6) but can be easily separated by having a single pectoral and 35 anal fin rays (vs 12–17 pectoral and 36–44 anal fin rays); 64 total vertebrae including 28 abdominal + 36 caudal (vs 66–68 total, 34–41 abdominal + 25–35 caudal vertebrae).

*Gangaichthys* gen. nov. is most similar to *Pillaia*, having a similar number of vertebrae (total 64 vs 62–74 in *Pillaia*, abdominal 28 vs 26–29, and caudal 36 vs 35–46); number of fin rays (dorsal 37 vs 34–44 and anal 35 vs 33–45). However, both these genera can be easily diagnosed from each other on the basis of non-overlapping pectoral (1 vs 6–11) and caudal (6 vs 8–12) fin ray counts.

##### Etymology.

From the Sanskrit *Ganga* for the Ganga or Ganges River and the Greek word *ichthys* meaning fish. Gender masculine.

#### Gangaichthys indonepalicus sp. nov.

Taxon classificationAnimalia

30A5A73B-7E8D-5C44-8F9A-2D83AC7E88E3

https://zoobank.org/4E1E5F4C-61B8-4E98-8917-D3CABF5976F3

Figs 2, 3, 4; Tables 2, 3

##### Type material examined.

***Holotype***. • NRC-AA-9519, 40.6 mm SL; India: Bihar State: Pashchim Champaran District: near Balgangwa, Akshay Khandekar, 5 January 2025. ***Paratype***. • NRC-AA-9695, 36.5 mm SL (damaged), same data as holotype.

##### Referred material.

• NRC-AA-9696, fragmentary, same data as holotype except collected from ~ 900 m N, 7 January 2025.

##### Diagnosis and comparative remarks.

The diagnosis for the new species and comparative remarks against the monotypic *Bihunichthys*, *Garo*, *Nagaichthys*, *Pillaiabrachia* are provided under the description of the genus above. *Gangaichthys indonepalicus* sp. nov. is further distinguished from other chaudhuriids in having 28 abdominal vertebrae (vs 34 abdominal vertebrae in *Chendol lubricus* and 39–41 in *Chendol keelini*); 36 caudal vertebrae (vs 42–45 caudal vertebrae in *Chaudhuria caudata*, 42–47 in *Chaudhuria fusipinnis*, 46–49 in *Chaudhuria ritvae*, 35 in *Chendol lubricus*, 25–28 in *Chendol keelini*, and 44–46 in *Pillaia kachinica*); 64 total vertebrae (vs 70–74 total vertebrae in *Chaudhuria caudata*, 71–74 in *Chaudhuria fusipinnis*, and 74–76 in *Chaudhuria ritvae*); 37 dorsal fin-rays (vs 39–42 dorsal fin-rays in *Chaudhuria caudata*, 44–48 in *Chaudhuria fusipinnis*, 40–43 in *Chaudhuria ritvae*, 43–45 in *Chendol keelini*, and 42–44 in *Pillaia kachinica*); 35 anal fin-rays (vs 39–43 anal fin-rays in *Chaudhuria caudata*, 44–48 in *Chaudhuria fusipinnis*, 41–44 in *Chendol keelini*, and 44–45 in *Pillaia kachinica*); six caudal fin-rays (vs 8–9 caudal fin-rays in *Chaudhuria caudata*, eight in *Chaudhuria ritvae*, 8–11 in *Pillaia indica*, and 10–12 in *Pillaia kachinica*); a single pectoral fin-ray (vs 7–9 pectoral fin-rays in *Chaudhuria caudata*, 6–10 in *Chaudhuria fusipinnis*, 7–10 in *Chaudhuria ritvae*, 12–14 in *Chendol lubricus*, 14–17 in *Chendol keelini*, 6–9 in *Pillaia indica*, and 10–11 in *Pillaia kachinica*); the absence of anterior spinelike projection on neural arch (vs presence of spinelike projection on neural arch in front of neural spine in *Chaudhuria caudata*, *Chendol keelini* and *Pillaia indica*); a single fused hypural (vs hypural divided into two in *Chaudhuria caudata*, and *Pillaia indica*).

##### Description of the holotype.

Body elongate, eel-like (Fig. 2A–C), greatest depth at dorsal-fin origin, 26.7× in SL. Dorsal and ventral profile more or less straight in abdominal and anterior caudal region, after which body tapering towards tail. Body anteriorly round in cross section up to anus, becoming increasingly laterally compressed posteriorly. Head elongate, cylindrical, 7.7× in SL; eyes reduced, covered by skin (Fig. 2D, E); mouth terminal, snout short, rounded, its length contained 3.5× in head length; tube of anterior nostril short, situated at tip of snout, projecting slightly from it; posterior nostril a fairly large opening, partially covered by skin flap (Fig. 2D). Lips fleshy, well-developed on upper and lower jaws; rictus situated in front of vertical through anterior margin of posterior nostril (Fig. 2D–F). Postorbital part of head slender, with six long branchiostegal rays supporting prominent gill membrane partially covering base of pectoral fin; gill opening restricted to ventral and ventrolateral side of head (Fig. 2F). Scales and lateral line canals absent, therefore myomeres and myosepta along body visible under microscope (but not clearly visible in Fig. 2B). Dorsal, anal, and caudal fins confluent. Dorsal-fin rays 37. Anal-fin rays 35. Caudal-fin rays 3+3. Pectoral-fin rays 1. Pelvic fin absent. Total number of vertebrae 64, comprising 28 abdominal and 36 caudal vertebrae.

##### Colouration (Fig. 3A, B).

Overall colour in life very pale and translucent in parts, with a few vascularised pink patches on gills and anal region, vertebral column visible as a pink line along caudal fin. Body, head and fins unpigmented, translucent; fin membranes translucent and fin rays whitish. In preservative, overall colouration faded to off-white, body slightly more opaque (Fig. 2A–C).

##### Osteology (Fig. 4).

The head skeleton is well ossified. The neurocranium is slender with the broadest part in the occipital region, tapering anteriorly. Nasal short and relatively broad with a posteriorly extending end contacting the dorsal surface of the lateral ethmoid. Lacrimal prominent with an expanded anterior part, narrow posteriorly and turning upward to form an ascending process which articulates with lateral end of lateral ethmoid. Paired frontals form anterior two-thirds of skull roof, suture between them distinct only posteriorly; the parietal, pterotic, supraoccipital, and epiotic form the posterior one-third of the skull roof. The parietal sutures anteriorly with posterior margin of the frontal and is incompletely divided anteriorly. The supraoccipital is bordered anterolaterally by the parietal, laterally by the pterotic, posterolaterally by the epiotic and exoccipital. Thus, the paired exoccipitals are separated by the supraoccipital. The foramen magnum is bordered by the supraoccipital dorsally and the exoccipital laterally and ventrally. The autosphenotic and prootic form the lateral wall of the neurocranium. The autosphenotic anteriorly sutures the narrow frontal descending lamina, dorsally contacting the parietal or the supraoccipital as the suture between these two bones is not clear, posteriorly sutures with the anterior margin of the pterotic. The prootic extends anteriorly. The anterior process (anterior extension) of prootic is evident. It is dorsally free from the autosphenotic. A large foramen enclosed by the dorsal margin of the prootic and ventral margin of the autosphenotic. As for the jaws and supsensorium, the premaxilla and maxilla are fused. The hyomandibula articulates with the neurocranium by two articulate heads: with the autosphenotic by the anterior one, with the pterotic by the posterior one. A large foramen pierces the low region of the hyomandibula. The metapterygoid has no anterior process to bridge it and the posterodorsal corner of the quadrate. No toothplate or teeth observed on the dorsal surface of hypobranchial 3. For the pectoral girdle, only an elongated cleithrum and a short supracleithrum are observed. No ossified scapula, coracoid, postcleithrum and pectoral ray are observed. There are totally 64 vertebrae including the ultimate compound ural centrum. All neural spines laterally narrow and tapering to a point posteriorly or dorso-posteriorly. No anterior spinelike projection present on neural arch. The first haemal spine appears at the vertebra 32. No ossified ribs are observed. The dorsal fin and anal fin are long and connected to the caudal fin by a membrane. There are 37 dorsal fin rays and 38 fin pterygiophore of dorsal fin. The first and second pterygiophores insert in the space between the neural spines of centra 26 and 27 and the first pterygiophore supports no ray, whereas the last pterygiophore inserts in the space between the neural spines of centra 61 and 62. There are 35 anal fin-rays and pterygiophores respectively, one pterygiophore supporting one ray. The first pterygiophore inserts in the space between the unclosed haemal spine of centra 28 and 29, whereas the last one inserts in the space between the haemal spine of centra 62 and 63. For the caudal skeleton and fin-rays, hypural is weakly ossified compared to vertebrae centra, and there are six caudal rays.

##### Variation.

We were able to only collect limited mensural data from the paratype, which notably varies from the holotype in size-standardised values for head length, snout length, and pectoral fin length; the smaller paratype having both an absolutely and relatively longer pectoral fin (Table 2).

##### Etymology.

The specific epithet is a toponym formed from India and Nepal, reflecting the distribution of the species in the Indo-Nepal border region. Suggested English common name is Indonepalese blind earthworm eel.

##### Distribution and natural history.

*Gangaichthys indonepalicus* sp. nov. is known from two closely-spaced localities in Balgangwa village, Pashchim Champaran District, Bihar State, India; and based on photographic records from Triveni in Nawalparasi District, Lumbini Province, Nepal; all within an 11-km straight-line distance. *Gangaichthys indonepalicus* sp. nov. appears to be exclusively subterranean as it has only been recovered from handpumps (borewells) reaching depths of up to ~ 10 m below the surface. The holotype and paratype (NRC-AA-9695) were collected from the same borewell after pumping out ~ 2000–2500 litres of water (Fig. 2C). The holotype emerged intact, while the other specimen sustained damage during pumping and died immediately. Subsequently, an additional specimen was collected from another handpump, ~ 1 km north of the type locality (Fig. 2C). This specimen was severely damaged and dead. We sampled a few nearby wells and ponds but could not find any additional specimens of the new species.

## Discussion

*Gangaichthys indonepalicus* gen. et sp. nov. is the first stygobitic and the fifth monotypic genus of Chaudhuriidae, extending the range of the family ~500 km westward. The new species is the northernmost representative of the family and the first from Bihar, India, and Nepal as well as the first subterranean fish from Indian alluvial aquifer systems and the Indo-Gangetic Plain. The hotspot for stygobitic fish in India is the lateritic aquifer system of Kerala in southern India with 11 species, including three loaches of the genera *Pangio* Blyth, 1860, four catfish of the genera *Horaglanis* Menon, 1950 and *Kryptoglanis* Vincent & Thomas, 2011, the snakehead *Aenigmachanna* Britz, Anoop, Dahanukar & Raghavan, 2019, and three synbranchids of the genus *Rakthamichthys* Britz, Dahanukar & Standing, 2020. Four synbranchids are known from other regions of India—one species each in northeast India and the northern Western Ghats of the genera *Rakthamichthys* and *Ophichthys* Swainson, 1839 (Anoop et al. 2019; Britz et al. 2014, 2019, 2020, 2021; Raghavan et al. 2022, 2023, 2025). Targeted sampling of stygobiont fish is required across India, with dangerously high levels of groundwater extraction and contamination likely affecting the subterranean fauna (Raghavan et al. 2021).

*Gangaichthys indonepalicus* gen. et sp. nov. shares the following derived characters with other chaudhuriids (after Britz and Kottelat 2003): presence of a long membrane bone process on autosphenotic (Fig. 4C), a boomerang-shaped ectopterygoid with a long preorbital extension (Fig. 4E), an anterior process of membrane bone of the metapterygoid (Fig. 4E), and a ventromedian keel on the first vertebra (Fig. 4C). It has a single hypural which Britz (2016) considered a derived character (the plesiomorphic state is two hypurals, seen in *Pillaia*, *Pillaiabrachia*, and *Chaudhuria*). *Gangaichthys indonepalicus* gen. et sp. nov. is otherwise a typical stygobiont, with reduced eyes and lack of melanin, and an elongate and highly miniaturized body (Wilkens and Strecker 2017; Borowsky 2018; Britz et al. 2019). Though no ossified ribs were observed, the lack of contrast agents precludes the detection of weakly ossified or chondrified structures typical of miniaturized subterranean fishes. As we had only one intact specimen, we were unable to conduct further scans. Species in the Chaudhuriidae are small to minute in size with a number of reductive characters linked to miniaturization and many live burrowing in the substrate of slow-flowing or stagnant waterbodies, but this is the first truly subterranean species (Kottelat and Lim 1994; Britz and Kottelat 2003; Britz 2016). Similar convergent adaptations to subterranean life are observed across numerous phylogenetically distant stygobitic fish lineages and do not represent shared ancestry but relaxed or directional selection in lightless, low-resource environments (e.g. Dahanukar et al. 2023). Even after excluding the characters linked to a hypogean lifestyle, the combination of unique morphological characters clearly distinguishes the new genus and species from other members of the Chaudhuriidae, but a lack of comparative sequence data for the family prevents elucidation of generic inter-relationships and the position of the new genus within the family. This should be a priority for future work on the family and to confirm our recognition of *Gangaichthys indonepalicus* gen. et sp. nov. as a new genus. The new species is recovered as the well-supported sister taxon to *Chaudhuria caudata*; the two collectively sister to *Pillaia indica*. While *Gangaichthys indonepalicus* gen. et sp. nov. could be construed to be a member of an expanded *Chaudhuria*, this is unlikely given the long branch length and the number of distinct morphological characters that differentiate it from *Chaudhuria* spp.

Shrestha (2008) provisionally named a small (33 mm), blind, eel-like fish from an unspecified locality in Narayani, southern Nepal; without designating a type specimen or referring to any museum specimens—making *Neoanguilla nepalensis* a nomen nudum as per Art. 16.1 and Art. 16.4. (Froese and Pauly 2025). While the affinities of the taxon referred to as “*Neoanguilla nepalensis*” remain unclear, it is a small, blind eel-like fish from the same geographic region as *Gangaichthys indonepalicus* gen. et sp. nov. However, it is clear that *Gangaichthys indonepalicus* gen. et sp. nov. is not conspecific with “*Neoanguilla nepalensis*” as the former has considerably more dorsal (37 vs 26–28) and anal (35) fin-rays and lacks melanophores and lateral line (vs presence of melanophores and lateral line) (Shrestha 2008).

## Supplementary Material

XML Treatment for Gangaichthys

XML Treatment for Gangaichthys indonepalicus

## Additional information

### Artificial Intelligence (AI) use

The authors accept full responsibility for the content of the manuscript, including the disclosure of any use of AI.

No AI tools were used in the preparation of this manuscript.

### Author ORCIDs

A. Khandekar https://orcid.org/0000-0002-7956-089X

T. Thackeray https://orcid.org/0000-0002-9981-8763

Y. He https://orcid.org/0009-0004-8027-0760

A. Mohapatra https://orcid.org/0000-0003-3547-7039

I. Agarwal https://orcid.org/0000-0001-9734-5379

### Data availability

All of the data that support the findings of this study are available in the main text.
